# Multiscale Sample Entropy of Two-Dimensional Decaying Turbulence

**DOI:** 10.3390/e23020245

**Published:** 2021-02-20

**Authors:** Ildoo Kim

**Affiliations:** Department of Mechanical and Electrical Engineering, Konkuk University, Chungju 27489, Korea; ildookim@kku.ac.kr

**Keywords:** sample entropy, decaying turbulence, information content

## Abstract

Multiscale sample entropy analysis has been developed to quantify the complexity and the predictability of a time series, originally developed for physiological time series. In this study, the analysis was applied to the turbulence data. We measured time series data for the velocity fluctuation, in either the longitudinal or transverse direction, of turbulent soap film flows at various locations. The research was to assess the feasibility of using the entropy analysis to qualitatively characterize turbulence, without using any conventional energetic analysis of turbulence. The study showed that the application of the entropy analysis to the turbulence data is promising. From the analysis, we successfully captured two important features of the turbulent soap films. It is indicated that the turbulence is anisotropic from the directional disparity. In addition, we observed that the most unpredictable time scale increases with the downstream distance, which is an indication of the decaying turbulence.

## 1. Introduction

The traditional discipline of turbulence research focuses on the flow of energy in phase space. This tradition began when Kolmogorov first derived the famous four-fifth law in 1941, as a direct inference of the Navier–Stokes equation, to illustrate that the second moment of velocity is always transferred from the large scale to the small scale [1]. The tradition continued as Kraichnan found the discrepancy between two-dimensional (2D) and three-dimensional (3D) turbulence in 1963, characterized by the inverse energy cascade and the forward enstrophy cascade [2]. These results are not only the milestones of turbulence research but also the archetypes showing the importance of the flow of energy in this curriculum.

By that time, physicists acknowledged that the energy is information, and vice versa. In 1948, Shannon suggested the concept of information entropy and paved a way to measure disorder using a concept similar to the thermodynamic entropy [3]. In 1961, Landauer showed that deletion of information causes dissipation of heat [4], indicating that there is a thermodynamic cost of information. Inspired by the advancement of information theory, there have been efforts to shed new light on turbulence by considering it as a flow of information. There was a paucity of studies under this paradigm, with a notable exception by Aubry et al. [5] Later additions include investigations by Kim [6] and Cerbus and Goldburg [7,8] addressing the properties of turbulence as a set of information. In recent years, “data science” is considered as an independent discipline, and there is an increasing number of studies that treat fluid motion as data flow [9,10].

The current study is aligned with those to understand the turbulence in terms of information. Specifically, we pay attention to sample entropy. Sample entropy is a variant of Kolmogorov–Sinai entropy, designed for physiological time series. It was first introduced by Pincus [11] as approximate entropy, which was later modified to sample entropy by Richman and Moorman [12,13] to remove methodological pitfalls. Essentially, it quantifies the predictability of a time series by examining the number of instances that a certain sub-string, which is observed to be repeated throughout the time series, is followed by the same consequence. It is defined as a negative logarithm of a probability, therefore it ranges from 0 (most predictable) to infinity (most unpredictable). Examples of its application are the studies of Costa et al. [14,15], where they successfully identified heart disorders using heartbeat time series measurements. Other examples of usage include the quantification of complexity in weather forecasting [16], postural sway [17], stock market [18], and feature extraction [19,20].

The system of interest of this study is a flowing soap film channel. The soap film channel was developed as a scientific instrument in 1980s [21,22,23,24], and since then it has been widely used to study 2D hydrodynamics including 2D turbulence. Typically, the turbulence in soap films is quasi-2D, meaning that the flow exhibits characteristics of 2D turbulence [25], but the medium is slightly compressible [26]. Notable accomplishments using soap films include the measurement of energy spectrum in 2D decaying turbulence [27,28], the observation of inverse energy and forward enstrophy cascades [29], the investigations on intermittency [30,31], and the measurement of turbulent friction factors [32,33]. From these conventional spectral analyses, it has been answered what is the exponent of energy spectrum of turbulent flow, whether the flow is isotropic or not, etc.

The central question of the current study was whether the inferences of the classical approach to turbulence can be obtained when we use the information theoretical approach. For the purpose, we applied sample entropy analysis to the velocity fluctuation measurements acquired from turbulent soap film flows. As discussed below, these velocity fluctuations were measured in the form of time series. Depending on the direction of the fluctuation, longitudinal or transverse, and the downstream location of measurement in the channel, we measured 42 time series for the analysis.

The answer to the central question is partly positive. First, the calculation of the sample entropy shows a clear disparity between the two directions of velocities, thereby indicating that our turbulence is anisotropic. Second, the results also show that the most unpredictable time scale is an increasing function of the downstream distance. This observation is consistent with our intuition, i.e., as turbulence decays, the system starts to exhibit longer autocorrelation time, and it increases the predictability at shorter time scales. These observations by themselves are not surprising at all. We already know that the turbulence in our setup is highly anisotropic and decaying, however we reached the same conclusion by only using an entropy approach without using any spectral analysis.

## 2. Method

### 2.1. Experimental Setup

**Figure 1:**
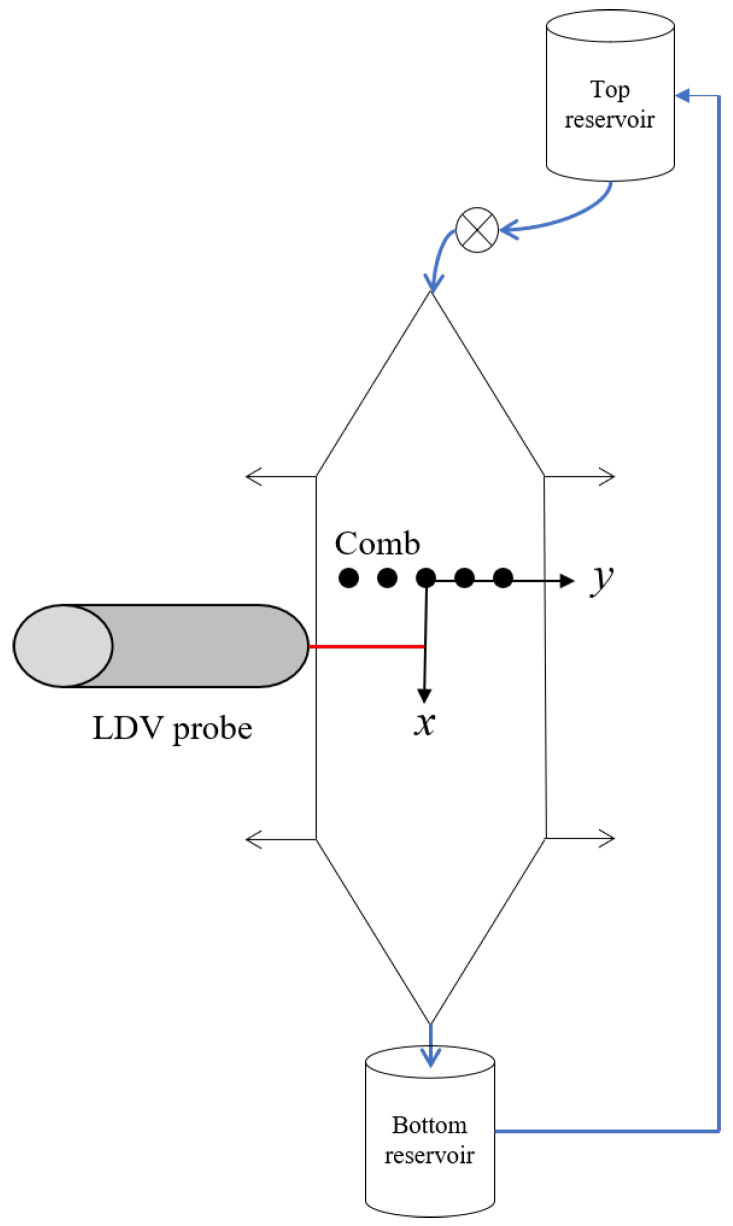
Experimental setup. Using a comb, we created a turbulent flow on a flowing soap film channel. The velocity field was measured at several locations downstream of the comb using laser Doppler velocimetry.

The experiments were conducted in a vertical soap film channel, as shown in Figure 1 and discussed in the literature [24,34]. Briefly, the soap film channel consists of two flexible nylon wires connected to two reservoirs at the top and bottom. To create a soap film, we first opened a valve between the top reservoir and the channel and established a flow of soap solution along two wires that are initially intact. Next, we pulled the wires apart from each other, and then a flow of soap film was created between the two wires. The soap solution was collected at the bottom reservoir and pumped back to the top reservoir. By recycling the soap solution, the flow of the film can last, in principle, indefinitely. Our soap solution was a 2:98 mixture of liquid detergent (Dawn, P&G) and deionized water, and the kinematic viscosity of the soap solution was approximately 0.13 ${\mathrm{cm}}^{2}/s$.

The soap film was quasi-2D as its thickness was much thinner than other dimensions. The length of the channel was approximately 1.5 m, and the width *W* of the channel was adjustable. For this study, we used two different soap films: a narrow film of W=2 cm, and a wider film of W=8 cm. In both cases, the thickness of the film was not measured directly, but we estimated it to be less than $10^{-5}$ m, based on the previous characterization of similar systems [26,34].

To make a turbulent flow on the soap film, we placed a comb approximately 0.5 m away from the top of the channel. At this location, the flow speed reached the terminal velocity and did not vary significantly. The mean flow speed *U* was approximately 1.5 m/s for both narrower and wider films. The comb’s teeth were the cylinders of diameter 0.001 m, each separated by 0.002 m. For the convenience of ensuing discussion, we set a coordinate system such that *x* is longitudinal and *y* is transversal to the flow direction. The origin of the coordinate system is located at the comb (see Figure 1). The definition of the coordinate system trivially indicates that the flow is laminar for x<0 and turbulent for x>0. There is no forcing other than the comb, and therefore the flow exhibits the characteristics of decaying turbulence.

We then measured the velocity field of the turbulent flow using laser Doppler velocimetry (LDV, manufactured by TSI scientific). We seeded the soap solution with polystyrene beads, whose diameter was $10^{-6}$ m, and we placed the LDV probe at several locations downstream of the comb, i.e., at (*X*, 0), where the value of *X* is summarized in Table 1. Because LDV measures the velocities of passive particles (polystyrene beads) in the flow, the sampling is rather random. In our experiments, the sampling rate varied from 9000 to 23,000 Hz with an average of 20,000 Hz. For each run, the measurement was performed for 40 s, and 800,000 data were acquired.

To summarize, our experiments produced a set of measurements of the velocity field of 2D decaying turbulence in the form of time series. These time series were collected for two different soap films at several different downstream distances for two components. For clarity of discussion, we denote a time series as $v_{\left(X,i\right)}^{N}\left(t\right)$, where *N* denotes the case number in Table 1, *X* denotes the downstream distance at which the LDV probe was placed, and *i* is either “*l*” (longitudinal) or “*t*” (transversal). When there is no possibility of confusion, indices may be omitted.

### 2.2. Data Preparation

**Figure 2:**
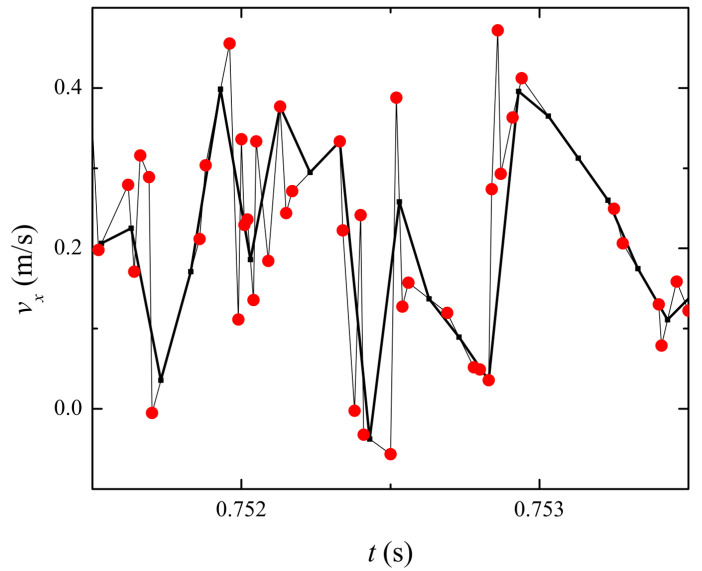
Example of raw (circles) and interpolated time series (thick lines). Laser Doppler velocimetry measured the flow velocity at random times. Therefore, we re-sampled the data using Equation (1) at 10,000 Hz .

Before performing the main analysis, the raw data from the measurements must be pre-processed. First, based on the nature of LDV, the velocity measurements were not acquired at an even time interval. As an example, we present a typical raw time series directly measured from LDV in Figure 2 with closed circles. It is clearly noticeable that the data are densely populated around 0.0752 s. However, they are sparsely populated around 0.0753 s. To remove the sampling irregularity, we linearly interpolated the data based on the uniform sampling time. Formally, for any integer *n*, we calculated the interpolated time series as follow:

(1) $$v\left(t_{n}\right)=v\left(t_{a}\right)+\frac{v\left(t_{b}\right)-v\left(t_{a}\right)}{\left(t_{b}-t_{a}\right)}\left(t_{n}-t_{a}\right).$$

where $t_{a}$ and $t_{b}$ ($t_{a}<t_{b}$) denote the abscissae of the two nearest data of $t_{n}=nt_{0}$. In our pre-processing, we used $t_{0}=0.1$ ms, i.e., the data were re-sampled at 10,000 Hz. The resulting time series correspond to v(n)={v(0),v(1),⋯,v(N−1)}, where *N* is 400,000. Second, the interpolated time series was normalized so that the mean is 0 and the standard deviation is 1. Each entry v(n) of the interpolated time series was substituted by v(n)−<v>/σ[v], where the mean corresponds to <v>=(1/N)Σv(n) and standard deviation corresponds to $\sigma \left[v\right]=\sqrt{\left(1/N\right)\Sigma {v(n)-<v>}^{2}}$.

Once the pre-processing was completed, the time series was ready for the main analysis. For such uniformly sampled and normalized time series, we introduced the coarse-graining factor α. Using this dimensionless integer, we generated a coarse-grained time series $\left\{v^{\left(\alpha \right)}\left(0\right),v^{\left(\alpha \right)}\left(1\right),\cdots ,v^{\left(\alpha \right)}\left(N/\tau -1\right)\right\}$ from the original time series {v(0),v(1),⋯,v(N−1)} via local and non-overlapping averaging. Therefore, each entry of the coarse-grained time series is as follow:

(2) $$v^{\alpha }\left(n\right)=\frac{1}{\alpha }\sum_{j=0}^{\tau -1}v\left(n\alpha +j\right).$$

We note that coarse-graining reduces the length of the time series. For example, for the original time series of length 400,000, the coarse-grained time series with α=4 corresponds to a length of 100,000. In addition, the time series was normalized only once at the initial preparation stage. Time series were not normalized in each coarse-graining process, and, therefore, the standard deviation decreases with α.

### 2.3. Multiscale Entropy Analysis

Once the data preparation process was completed, we calculated the sample entropy of the time series of the turbulent flow. The concept of sample entropy was detailed by Costa [14]. Briefly, sample entropy is designed to quantify the predictability of a time series. Formally, it is a negative logarithm of a probability wherein the posterior is repeated for a given certain prior. Let us consider a subset of a time series whose length is *m*, that is, $s_{m}\left(n\right)=\left\{v\left(n\right),v\left(n+1\right),\cdots ,v\left(n+m-1\right)\right\}$, and assume that this subset is repeated *A* times throughout the entire time series. This means that there are (A−1) more choices of non-zero integer *l* such that subsets $s_{m}\left(n+l\right)=\left\{v\left(n+l\right),v\left(n+l+1\right),\cdots ,v\left(n+l+m-1\right)\right\}$ are equal to $s_{m}\left(n\right)$.

From a computational perspective, it is difficult to judge if $s_{m}\left(n\right)=s_{m}\left(n+l\right)$ when the data are processed in double precision. Therefore, we set a tolerance range *r*, and, if the difference between each element of two subsets as less than *r*, then they were considered as identical. Formally, $s_{m}\left(n\right)=s_{m}\left(n+l\right)$ if |v(n+k)−v(n+l+k)|<r for all k<m.

Now, we examined the probability at which *A* identical subsets are followed by the same value. We determined the number of instances that the inequality |v(n+m)−v(n+l+m)|<r is satisfied, and let *B* be the number of such cases. The sample entropy is defined as a negative logarithm of conditional probability p=B/A as follow:

(3) S=−lnp.

In multiscale sample entropy analysis, we calculated the sample entropy with respect to the coarse-graining factor α. In general, the probability *p* depends on *m*, *r*, and α. However, following the widely accepted convention, we fixed m=2 and r=0.15. This setting has been frequently used in extant studies [14,15,16], and we used the convention for future referencing.

**Figure 3:**
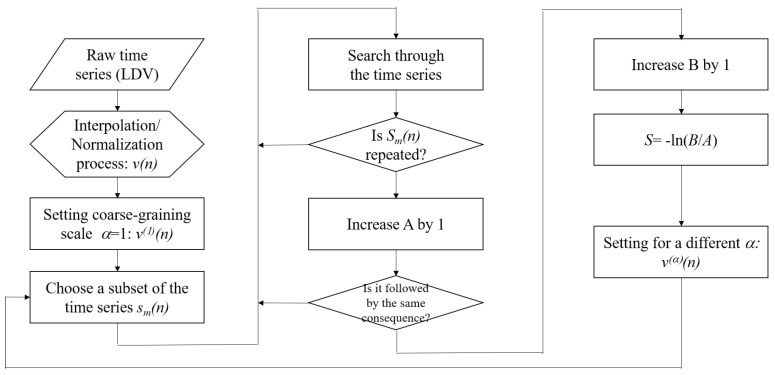
A flowchart to calculate the sample entropy.

Finally, for an intuitive discussion, we summarize the initial data preparation and the subsequent multiscale entropy analysis in the flowchart in Figure 3. In addition, we converted the coarse-graining factor α to the coarse-graining time scale τ by using the relation $\tau =\alpha t_{0}$. Therefore, S=S(τ).

## 3. Results and Discussion

### 3.1. Directional Disparity

**Figure 4:**
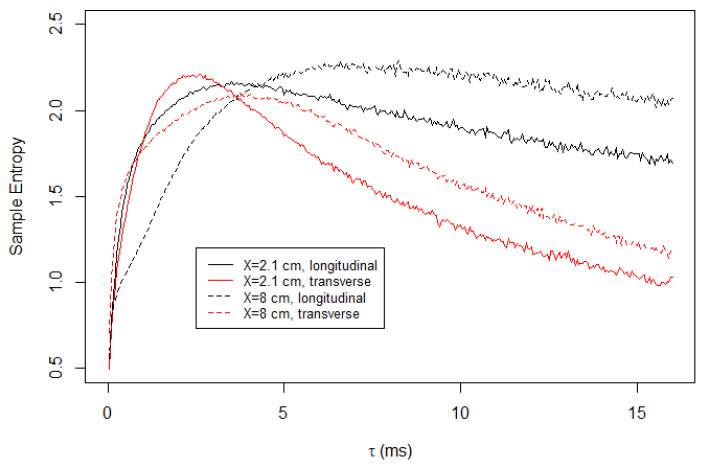
Sample entropy calculated for turbulent flow in Soap Film 1. At shorter time scales, the transverse component is more unpredictable. However, at a longer time scale, the opposite is true. We note that the sample entropy naturally decreases with τ because the coarse-grained time series are not normalized. The time series is normalized only once for α=1.

Figure 4 shows the result of the multiscale entropy analysis for the longitudinal and transversal components of the velocity time series, measured at two different locations on Soap Film 1. The results from the longitudinal and transversal components of the velocity measurements are qualitatively different. At shorter time scales, the transversal components of both measurement locations, X=2.1 cm and X=8.0 cm, yield a higher value of *S* than their longitudinal counterparts. However, the trend is reversed at a longer time scale. As τ increases, the sample entropies of the transverse component decrease rapidly. Conversely, the sample entropies of the longitudinal component do not decay as much as the transversal component.

The result in Figure 4 indicates that the turbulence in the flowing soap films is anisotropic. The discrepancy between the longitudinal and transverse components at the short time scale is attributed to the fact that the initial forcing is unidirectional. Using the diameter of the tooths of the comb, D=2 mm, and the mean flow speed, U=1.5 m/s, we estimated the Reynolds number as approximately 2100, and at this Reynolds number the vortex shedding frequency is approximately 150 Hz. This corresponds to 6.7 ms and is roughly consistent with our observation that the sample entropies are maximized at a few milliseconds.

**Figure 5:**
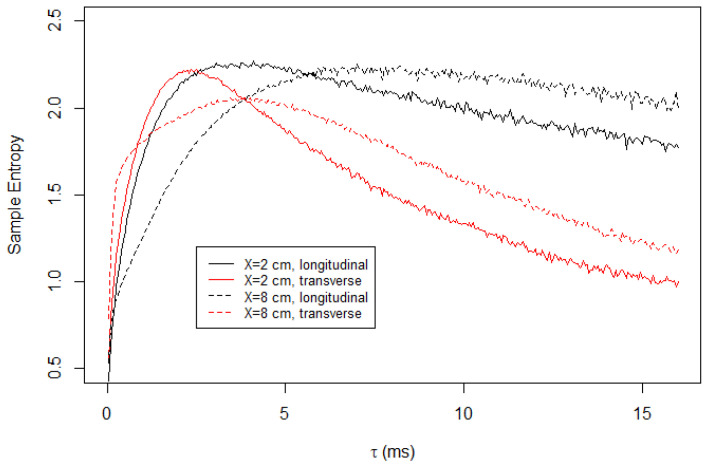
Sample entropy calculated for turbulent flow in Soap Film 2.

The substantial unpredictability of the longitudinal component at larger τ is puzzling. One hypothesis for this is that the friction between the channel walls or the air act as forcing to the turbulence. However, we reject this hypothesis because the sample entropy calculated for Soap Film 2, which is much wider than Soap Film 1, exhibits the same feature, as presented in Figure 5. If the hypothesis were true, then the wider film would show significantly less unpredictability at large τ because the forcing only affects locally.

### 3.2. Change over Downstream Distance

**Figure 6:**
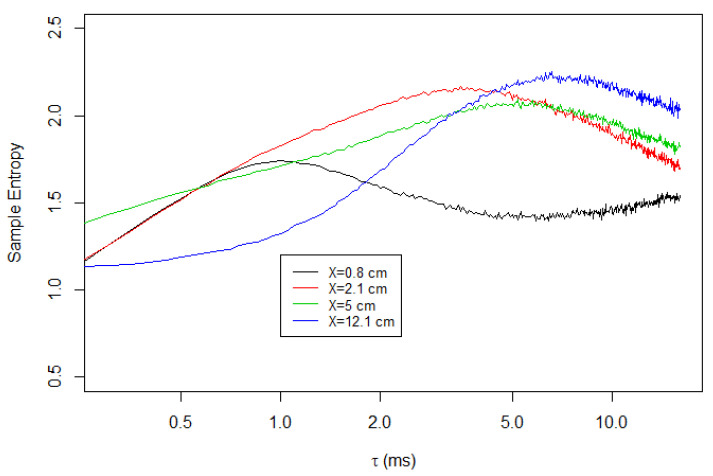
Multiscale sample entropy of the longitudinal velocities of Soap Film 1.

Figure 6 shows the multiscale entropy analysis of the longitudinal velocity of Soap Film 1, for the measurements at various downstream locations. We note that the time axis is logarithmic for a better presentation. The figure shows that S(τ) initially increases with τ. However, it decreases when τ is large enough. Therefore, S(τ) is maximized at a certain coarse-graining time scale $\tau_{max}$, and we find that $\tau_{max}$ increases as the measurement location *X* increases. For example, $\tau_{max}∼1$ ms at X=0.8 cm and $\tau_{max}∼3.5$ ms at X=2.1 cm. At x=12.1 cm, $\tau_{max}$ is as large as 6.5 ms.

**Figure 7:**
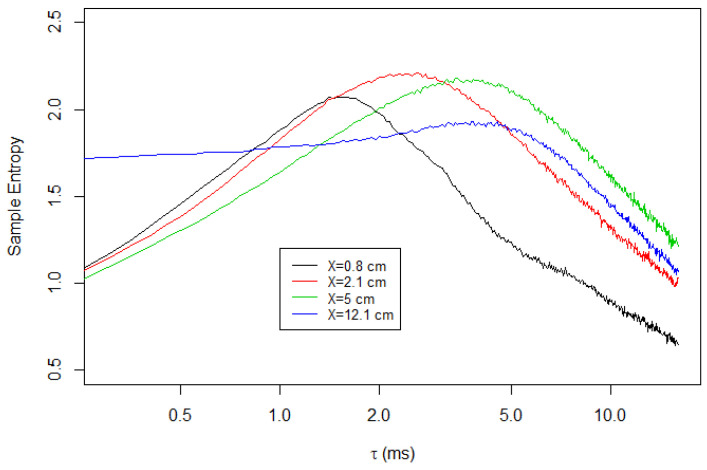
Multiscale sample entropy of the transversal velocities of Soap Film 1.

Figure 7 shows the same calculation using the transversal component. Here, peak shifting is observed in the transversal component in a manner similar to the longitudinal component. While the height of the peaks varies, the location of the peak moves toward the higher τ as the measurement location moves downstream.

**Figure 8:**
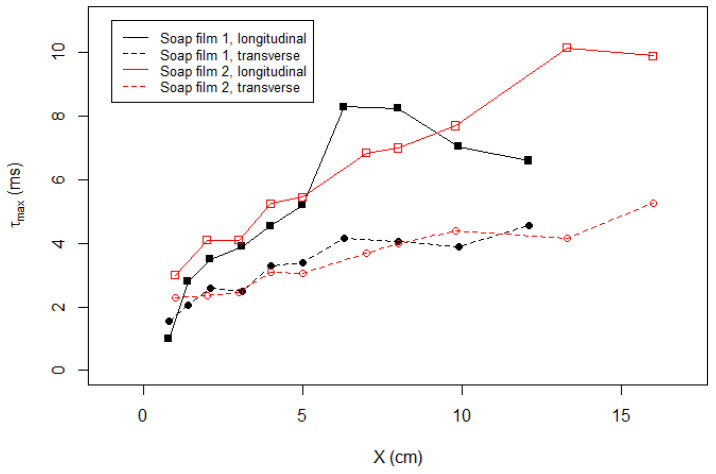
Coarse-graining time maximizing the sample entropy analysis, $\tau_{max}$, is plotted with respect to the downstream distance *X*.

In Figure 8, we plot our measurement of $\tau_{max}$ with respect to the downstream distance *X*. For Soap Film 1 or 2 and longitudinal or transverse component, $\tau_{max}$ generally increases with *X*. Physically, $\tau_{max}$ is the time scale at which the time series is most unpredictable, and it can be interpreted as the dissipation of turbulent energy at small scales. In soap film flows, we assume the frozen turbulence hypothesis. Therefore, the downstream distance is equivalent to time. As the turbulence moves downstream, or as time elapses, the finer structure decays, and only large-scale disturbance survives. From this perspective, our observation of peak shifting in Figure 6 and Figure 7 is considered as an indication of the decaying turbulence.

Finally, the current result raises an interesting question whether the entropic analysis can enhance the current understanding of turbulence. The related open problems include the issue of finite dissipation [35] and coherent structures [36]. The answers to these questions are beyond the scope of current work, but they are noteworthy for future studies.

## 4. Conclusions

In summary, we present a multiscale sample entropy analysis that was performed for the decaying turbulence on soap film flows. Without conventional analysis of turbulence such as the power spectrum, the multiscale sample entropy analysis allowed us to characterize the system of interest. First, the disparity between the results using the longitudinal and transversal velocity components revealed the anisotropy of the system. Second, based on the observation that the most unpredictable time scale increases with the downstream distance, it is inferred that the characteristic frequency of the turbulence decreases. This indicates that the small-scale disturbance is dissipated by the action of viscosity, and therefore the turbulence is indeed decaying.

## Figures and Tables

**Table 1:** Experimental conditions.

Case	Channel Width	Measurement Location (*X*, in cm)
1	2.1 cm	0.8, 1.4, 2.1, 3.1, 4.0, 5.0, 6.3, 8.0, 9.9, 12
2	7.7 cm	1.0, 2.0, 3.0, 4.0, 5.0, 6.0, 7.0, 8.0, 9.8, 13, 16

## Data Availability

Data may be available upon request.
